# Comparison of lingual mucosa and buccal mucosa grafts used in inlay urethroplasty in failed hypospadias of pre-pubertal boys in a Chinese group

**DOI:** 10.1371/journal.pone.0182803

**Published:** 2017-08-17

**Authors:** Jin Hongyong, Chen Shuzhu, Wu Min, Ye Weijing, Liu Yidong

**Affiliations:** 1 The Urology Center of XinJiang Uygur Autonomous Region People's Hospital, Urumqi, China; 2 Department of Urology, Ren Ji Hospital, School of Medicine, Shanghai Jiao Tong University, Shanghai, China; Assiut University Faculty of Medicine, EGYPT

## Abstract

**Objective:**

The purpose of this study was to compare the outcomes of the buccal mucosa and lingual mucosa used in children who received multiple failed hypospadias surgeries.

**Method:**

We conducted a retrospective study of 62 children who received buccal or lingual mucosa graft urethroplasty in our hospital between 2012 and 2015. The ages ranged from 3.5–11 y. All cases included multiple failed hypospadias procedures, and the subjects received previous operations 2–3 times. All patients underwent one-stage operations. Thirty-three cases were treated with lingual mucosa grafts. The patient ages ranged from 3.5 to 11 y (median 7.5 y), and they had previous operations 2–3 times (mean 2.8±0.7). Grafts ranged from lengths of 2–6 cm (mean 5.1±0.46 cm) and widths of 0.5–1.5 cm (mean 1.2± 0.16 cm). Our follow-up was 5 to 12 m (mean 8.3±1.2 m). Twenty-nine cases were treated with buccal mucosa grafts. The patient ages ranged from 4 to 9.2 y (median 7.0 y), and they had previous operations 2–3 times (mean 2.5±0.2). Grafts ranged from lengths of 2–5.3 cm (mean 4.9± 0.28 cm) and widths of 0.5–1.5 cm (mean 1.0±0.11 cm). Our follow-up was 5 to 12 m (mean 7.9±0.5 m). The results were tested with SPSS 18.0. The rates of complications were compared by a chi-square test, and pre-operative conditions were compared by t test.

**Results:**

For the outcomes of the two groups, there was no significant difference between the groups in terms of age, preoperative surgery time, and the length and width of the grafts (p>0.05). For the lingual mucosa graft group, fistula: 2/33 (6.0%), stricture: 1/33(3.0%), ventral curvature: 2/33(6.0%), complications: 5/33(15.0%), success rate: 28/33(84.8%), Hose score: 14.34±0.95, peak flow: 6.5 ml/s-12.0 ml/s, and mean peak flow: 9.3±0.4 ml/s. For the buccal mucosa graft group, fistula: 2/29(6.8%), stricture: 2/29(6.8%), ventral curvature: 1/29 (3.4%), complication rate: 5/29(17.0%), success rate: 24/29 (83.0%), Hose score: 14.28±1.03, peak flow: 6.5 ml/s-12.0 ml/s, and mean peak flow: 9.2±0.2 ml/s. There were no differences between the two groups for overall success, complication rates, peak flow, and the Hose scores(P>0.05).

**Conclusion:**

The lingual mucosal graft and the buccal mucosa graft both achieved good outcomes, and the lingual mucosa graft made up for the shortcomings of the buccal mucosa graft, which provided a reliable way to treat the multiple failed hypospadias surgeries in pre-pubertal boys.

## Background

The incidence of hypospadias is approximately 1/300, which has rapidly grown in recent years. Because surgeries are the only way to solve hypospadias, many studies of surgeries for the condition have been reported in the literature. We found different types of surgeries with different outcomes [1–3]. It is very challenging to complete penis reconstruction in patients who have had multiple procedures that failed. Failed hypospadias repair is often associated with penile skin loss and deficient local tissues, so other tissues are needed to complete the procedure. Based on the literature from Pubmed, a wide variety of grafts have been used for urethral reconstruction after failed hypospadias procedures [4]. Since Humby first reported using the buccal mucosa graft in 1941, the BMG has been widely accepted and has achieved satisfactory outcomes for patients who have had multiple failed hypospadias procedures[5, 6]. However, donor-site-related complications have been reported, such as the as numbness and tightness of the mouth, among other complications [7]. Additionally, patients who experienced failed hypospadias procedures often had long urethral defects and buccal mucosa grafts were not possible due to lack of available tissue. If buccal mucosa grafts are confronted with scarred tissues, BMG urethroplasty cannot be chosen again. Simonato et al were the first to report using tongue mucosa as an alternative donor site for urethroplasty in 2006, and their group had achieved good outcomes. Much literature has shown us that these techniques were effective. Yet, few reports have sought to compare outcomes of the BMG and LMG used in inlay graft urethroplasty in pre-pubertal Chinese boys. The aim of our study was to compare the two graft types and confirm the reliability of LMG in pre-pubertal boys from a Chinese population.

## Materials and methods

### Materials

We recruited sixty-two pre-pubertal boys who received buccal or lingual mucosa graft urethroplasty in our hospital between 2012 and 2015(S1 File). The patients’ ages ranged from 3.5–11 y. The measurements of the urethral meatuses of the LMG and BMG groups (Table 1) were identified in surgery. The inclusion criteria were: 1) two or more failed previous hypospadias repairs, and 2) penile skin that was no longer suitable for an onlay flap procedure. The exclusion criteria were: 1) ventral curvature >60 degrees identified by artificial erection in surgery, 2) physiological deficiency in the oral or lingual grafts, and 3) incomplete follow-up results or examinations. All patients underwent single-stage operations. Thirty-three cases were treated with lingual mucosa grafts, and twenty-nine cases were treated with buccal mucosa grafts. All surgeries were performed by 2 senior pediatric urologists (Ye weijing and Liu yidong). Written informed consent was obtained from the parents of all the patients and control subjects before the study began. Ethical approval was obtained from the Institutional Review Board of Renji Hospital, which was affiliated with the Shanghai Jiao Tong University School of Medicine

**Table 1:** The measurements of the urethral meatuses of the LMG and BMG groups identified in surgery. (P>0.05).

			Group		Total(n)
			LMG	BMG	
Position	distal	count	10	13	23
		position %	43.5%	56.5%	
		group %	30.3%	44.8%	
		total %	16.1%	21.0%	
	middle	count	13	8	21
		position %	61.9%	38.1%	
		group %	39.4%	27.6%	
		total %	21.0%	12.9%	
	proximal	count	10	8	18
		position %	55.6%	44.4%	
		group %	30.3%	27.6%	
		total %	16.1%	12.9%	
Total(n)		count	33	29	62
			

### Methods

**Fig 1:**
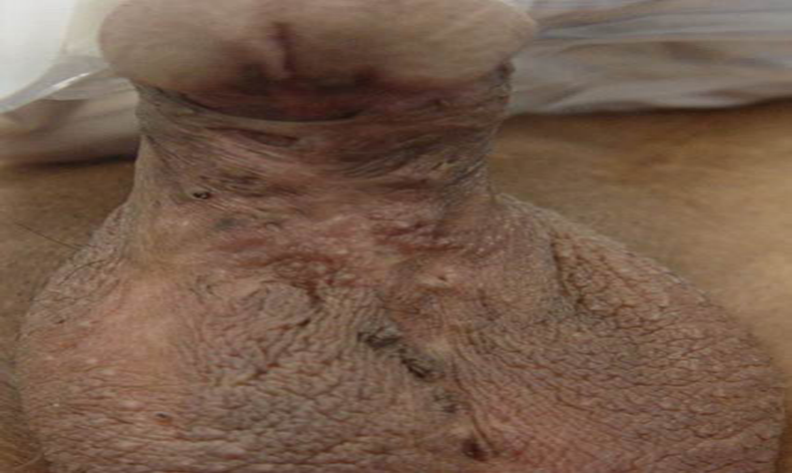
The appearance of the failed hypospadias.

**Fig 2:**
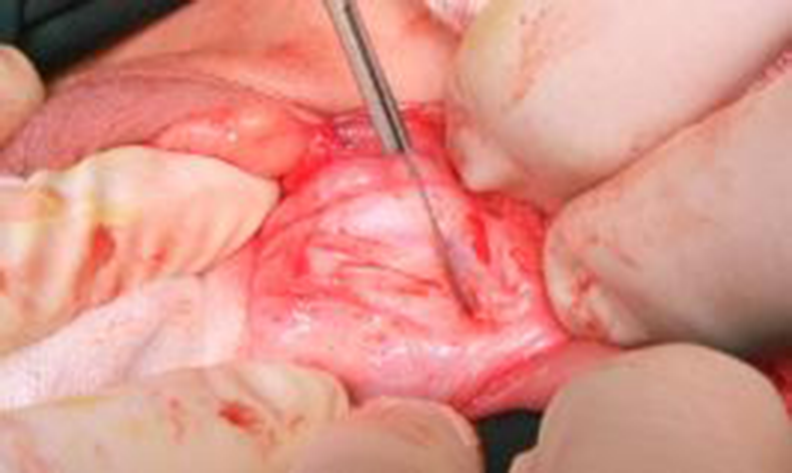
Removing the scar tissue and fistula.

The preparation of the LMG before the operation was similar to BMG urethroplasty. Because all of the 62 patients could have received either surgery, we assigned the two procedures at different periods. All patients were operated on general anesthesia with nasotracheal intubation. We documented the appearance of the failed hypospadias procedures (Fig 1). For the procedure, we attempted to maintain the original urethra and complete the penile skin degloving. Then, we removed the scar tissue and fistula (Fig 2). We opened the stricture of the urethra to a normal diameter. A deep midline relaxing incision was made in the urethral plate to the tunica albuginea if we were unable to tubularize because of strictures. If a ventral curvature existed due to a scarred urethral plate, we removed the inelastic ventral tissue. Artificial erection was used to confirm there was no chordee.

**Fig 3:**
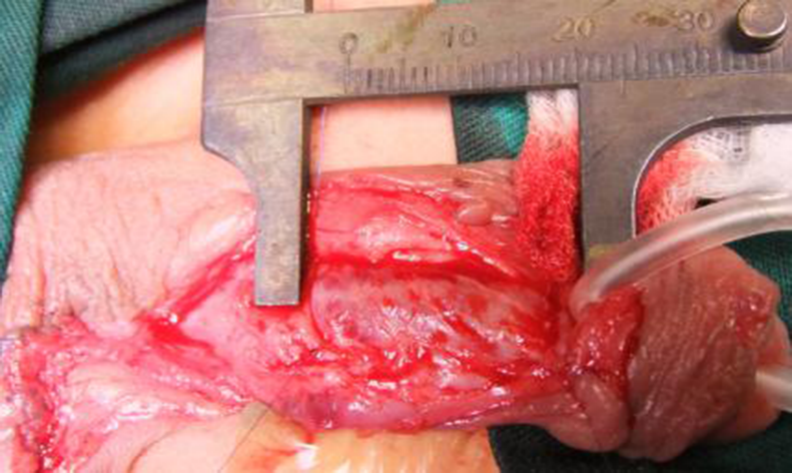
Measuring the urethral defect.

**Fig 4:**
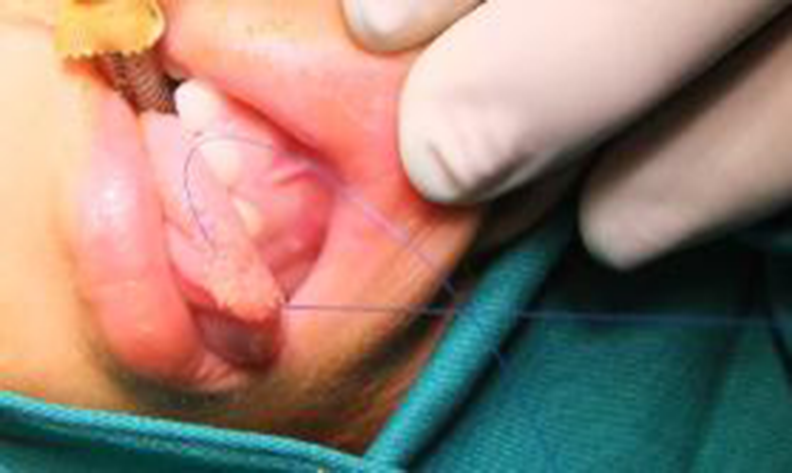
Exposing the ventrolateral surface of the tongue.

**Fig 5:**
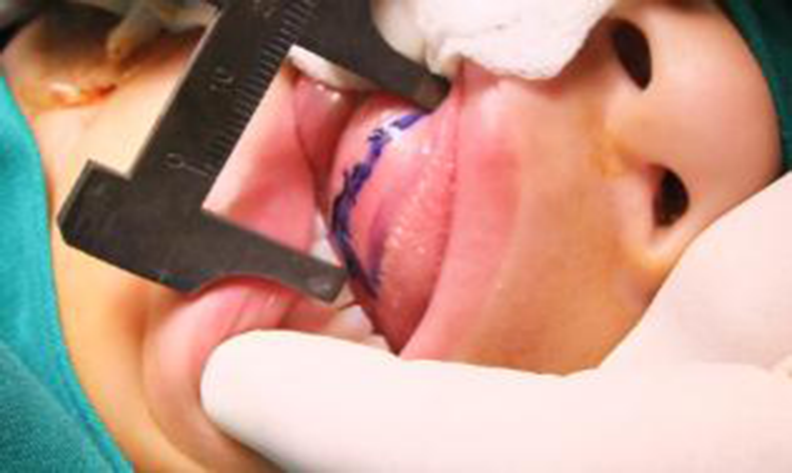
The graft is marked with a surgical pen on the ventro-lateral surface of the tongue.

**Fig 6:**
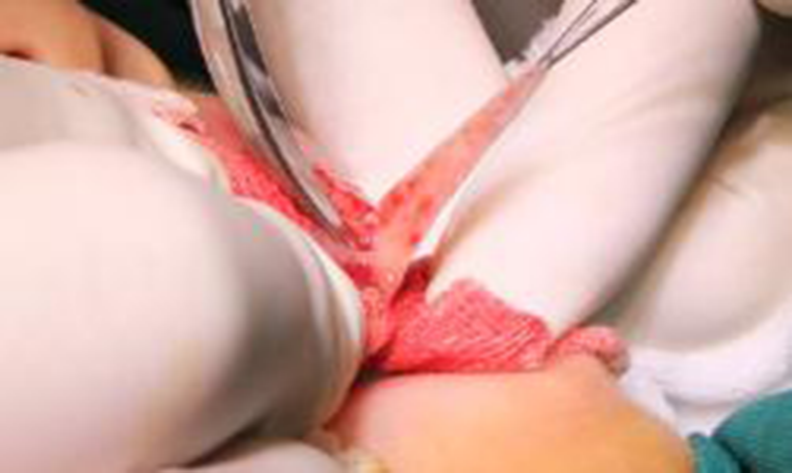
The graft is removed using sharp scissors.

Harvesting method for the lingual mucosa grafts: We measured the urethral defect (Fig 3), keeping in mind 15–20% shrinkage of the graft size. A 4–0 prolene stitch was passed through the apex of the tongue or grasped by a tongue forceps for traction outside of the mouth to expose the ventro-lateral surface of the tongue, which made it feasible for us to obtain the lingual mucosa (Fig 4). The graft was marked with a surgical pen on the ventrolateral surface of the tongue (Fig 5). The graft borders were incised using a scalpel along the marked line and then the graft was removed using sharp scissors (Fig 6).

**Fig 7:**
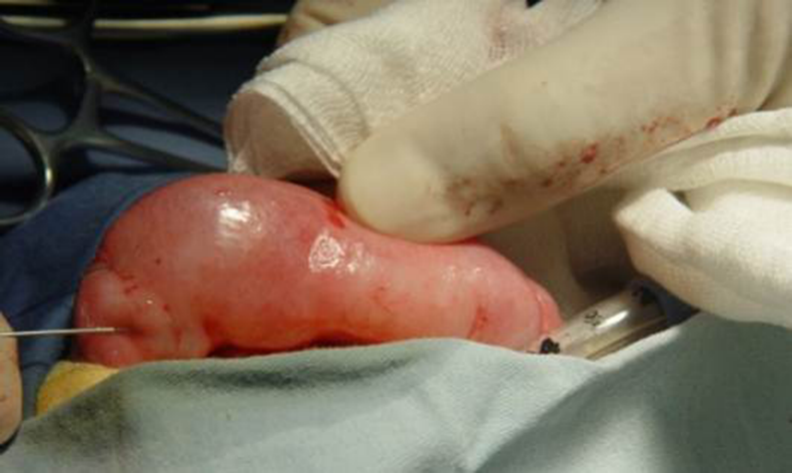
Harvesting method of the buccal mucosa grafts: The lower lip mucosa is fully exposed and nor-epinephrine (1:200,000) is applied before harvesting the graft.

**Fig 8:**
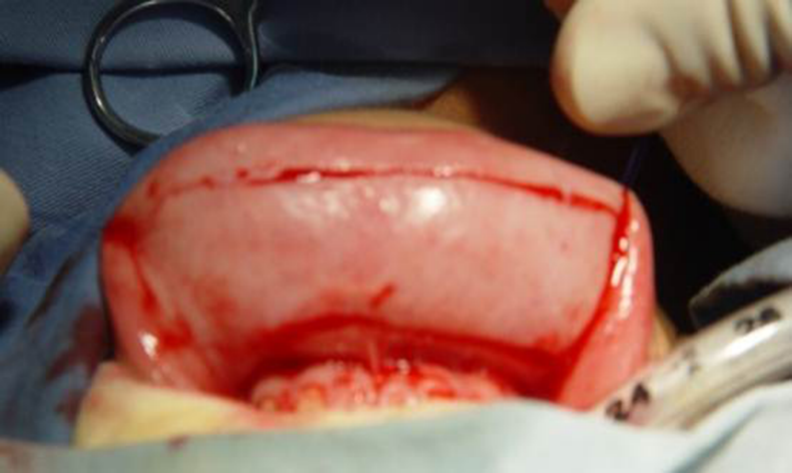
Harvesting method of the buccal mucosa grafts: The lower lip mucosa is fully exposed and nor-epinephrine (1:200,000) is applied before harvesting the graft.

Harvesting method for the buccal mucosa grafts: We measured the defect and fully exposed the lower lip mucosa. Nor-epinephrine (1:200,000) was applied to the area in both groups, and the graft was harvested (Figs 7 and 8).

**Fig 9:**
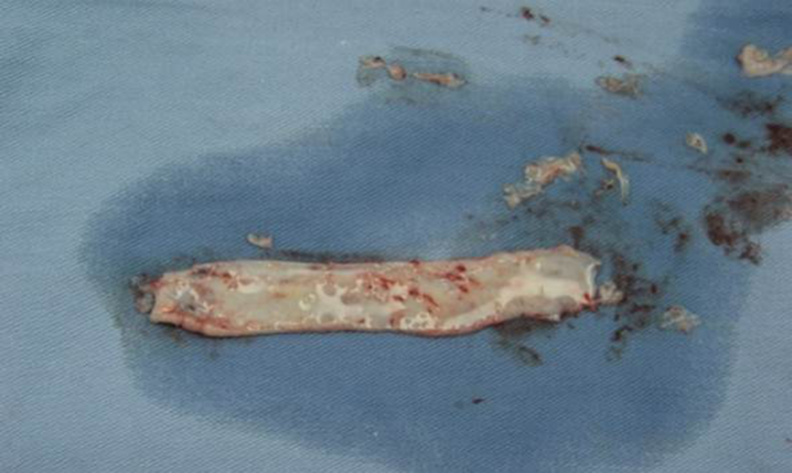
Graft defatting.

**Fig 10:**
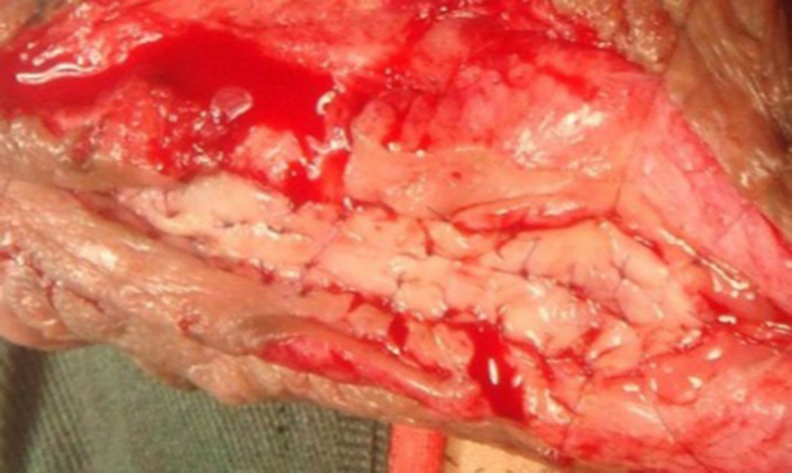
Making more quilting sutures and small incisions in the graft to avoid operative hematoma.

**Fig 11:**
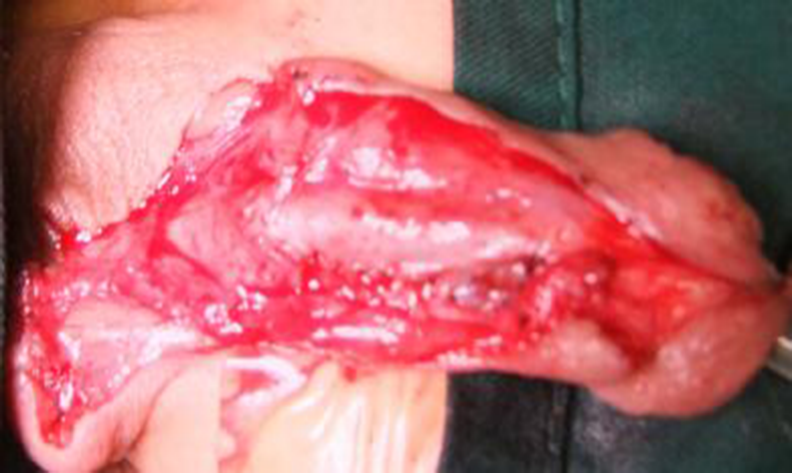
The neourethra is covered by a subcutaneous dartos patch.

**Fig 12:**
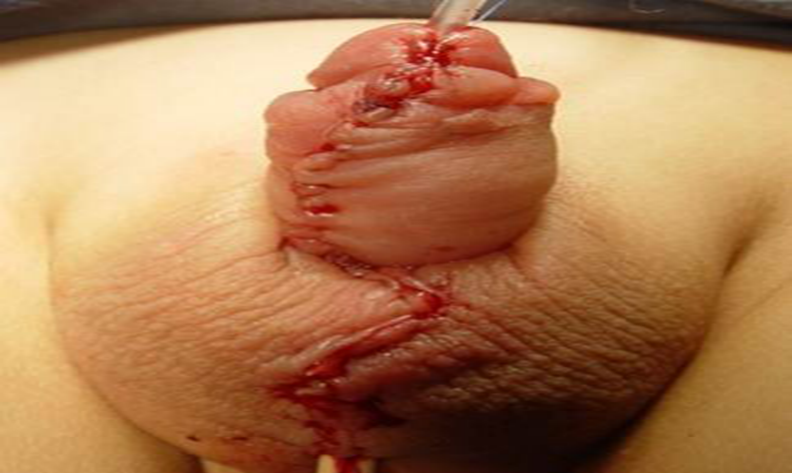
The glans and the penile skin are closed over the urethra at the end.

**Fig 13:**
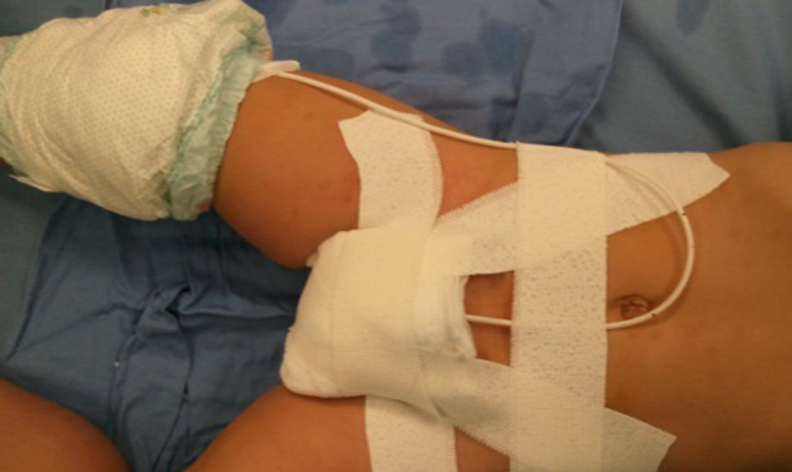
A pressure bandage is needed after surgery.

**Fig 14:**
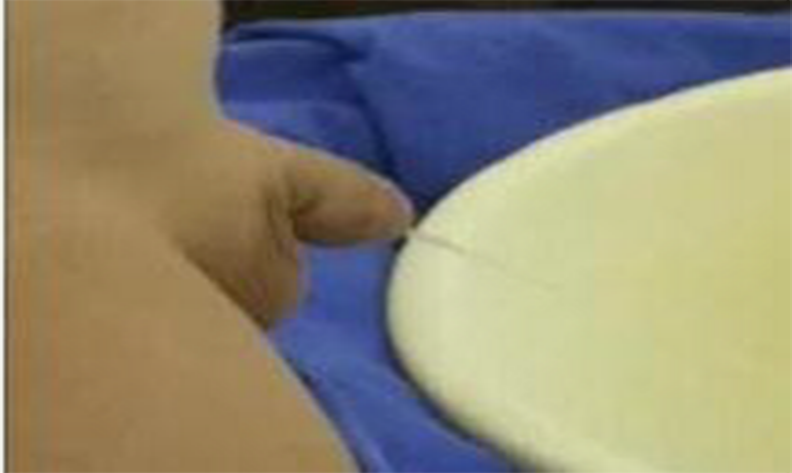
The follow-up appearance.

The graft was defatted (Fig 9). The donor site was examined for bleeding and closed with interrupted 5–0 polyglactin sutures. The graft was sutured into the incised urethral plate with 7–0 polyglactin. More quilting sutures and small incisions in the graft were made to avoid operative hematomas (Fig 10). The urethral plate was tubularized with 7–0 polydioxanone sutures in a continuous fashion over a 6–10 Fr catheter (which was selected based on the patient’s age or urethra size). The neourethra was covered by a subcutaneous dartos patch or some other tissues like tunica vaginalis if necessary (Fig 11). The glans and the penile skin were closed over the urethra at the end (Fig 12). A pressure bandage was needed after surgery (Fig 13). The appearance in the follow-up (Fig 14).

## Post-operative follow-up

The urethral catheter was removed after 3 weeks. For data analysis, our hospital used IBM SPSS version 18.0 for statistical analysis. The comparison of urethral meatus measurements and the outcomes was performed with a chi-square test, and P < 0.05 indicated that the differences were statistically significant. We assessed the outcome of the cosmetic results with the HOSE score table (designed by A.J.A. Holland in 2001 [8]). The scores and the follow-up periods of the groups were analyzed with SPSS 18.0, and the comparison of rates and pre-operative conditions were performed using a t-test with P<0.05 indicating statistically significant differences.

A successful outcome was defined based on the presence or absence of complications, including a fistula (which required an additional intervention), diverticula (which could affect the urine stream and require surgery), meatal stenosis/stricture (with stricture apparent on a urethrogram and requiring subsequent intervention), persistent ventral curvature, glans dehiscence and/or skin reoperation with some bleeding, and a loss of stent [9].

## Results

For the outcomes of the two groups, there was no significant difference between groups in terms of age, previous repairs, and the length and width of the grafts (p>0.05) (Table 2). For the lingual mucosal graft group, fistula: 2/33 (6.0%), stricture: 1/33 (3.0%), ventral curvature: 2/33(6.0%), complications: 5/33(15.0%), success rate: 28/33(84.8%), Hose score: 14.34±0.95, peak flow: 6.5 ml/s-12.0 ml/s, and mean peak flow: 9.3±0.4 ml/s. For the buccal mucosa graft group: fistula: 2/29 (6.8%), stricture: 2/29 (6.8%), ventral curvature: 1/29 (3.4%), complication rate: 5/29(17.0%), success rate: 24/29 (83.0%), Hose score: 14.28±1.03, peak flow: 6.5 ml/s-12.0 ml/s, and mean peak flow: 9.2±0.2 ml/s. There were no differences between the two groups for overall success, complication rates, the peak flow, and the Hose scores (P>0.05) (Table 3).

**Table 2:** The pre-operative conditions for the two groups.

Group	LMG	BMG	P
Age, y, Median, (range)	7.5(3.5–11)	7.0(4–9.2y)	P = 0.24
Previous repairs(n)	2.8±0.7	2.5±0.2	P = 0.05
Graft length (cm)	5.1±0.46	4.9±0.28	P = 0.18
Graft width(cm)	1.2±0.16	1.0±0.11	P = 0.07
Total(n)	33	29	

**Table 3:** The follow-up results for the two groups.

Group	LMG	BMG	P
Follow-up time (m)	8.3±1.2	7.9±0.5	P = 0.14
Complication	5/33(15.0%)	5/29(17.0%)	P = 0.66
Fistula	2/33(6.0%)	2/29 (6.8%)	P = 0.89
Stricture	1/33 (3.0%)	2/29 (6.8%)	P = 0.47
Ventral curvature	2/33(6.0%)	1/29(3.4%)	P = 0.62
Success	28/33(84.8%)	24/29(83.0%)	P = 0.66
The peak flow(ml/s)	9.3±0.4	9.2±0.2	P = 0.54
HOSE score	14.34±0.95	14.28±1.03	P = 0.82
Total (n)	33	29	

In our study, 10 cases were defined as failures based on the definition of success. Three patients were diagnosed with urethra strictures (one located at the junction of the urethral mucosa grafts, two at the urethral opening) based on the urethrography. All returned to normal after receiving dilation(once a week, 2-4weeks). Four patients with fistulas had received fistulous neoplasty. Three cases with ventral curvature (artificial erection >30 degree) were conducted with dorsal tunica albuginea plication based on the methods of Baskin (1994) [10]. No other complications arose.

## Discussion

Urethroplasty is the key procedure for treating hypospadias and different types of procedures have appeared over the years. Many patients have to face multiple surgeries due to failed procedures, and these patients typically develop problematic conditions, such as stricture, fistula, and ventral curvature [11, 12]. It is very challenging to perform the reconstructions for complex conditions. Many studies have been reported trying alternative materials for reconstructing the urethra. The most common material should be the prepuce flap[13] [14]. Li zc et al [15] used bladder mucosa grafts as a new alternative approach, and they have achieved success. Xu YM, et al [16] reported urethral reconstruction using colonic mucosa grafts for complex strictures, and they also have good results. However, these methods are rarely acceptable for treating patients with multiple failed hypospadias repairs. These cases are often associated with penile skin loss and deficient local tissues, which resulted from the damage to the prepuce flaps used for repair(s), and the colonic and bladder procedures are too complex. Since Humby et al first tried to apply buccal mucosa grafts to reconstruct the urethra in 1941, the buccal mucosa graft has emerged as a reliable and popular donor tissue for urethral substitution over the past few years. The advantages of BMG are easy harvesting, a thick epithelium, a thin lamina propria, a good blood supply with no hair and high survival [17]. These characteristics make BMG a popular substitution for urethral reconstruction.

However, this method is not suitable to patients with longer urethral defects, physiological deficiency of oral grafts or oral mucosa damage due to previous harvestings. Additionally, many scholars have reported that the buccal mucosa graft donor site has some complications, such as persistent perioral numbness, salivary changes, and difficulty in opening the mouth [7]. This dilemma has encouraged surgeons to find other approaches. Simonato et al [18] first reported the use of lingual mucosa as a substitute tissue for graft urethroplasty in 2006, and since then, many LMG reports have emerged with good results. Maarouf et al[19] reported 21 cases with the use of LMG (mean age 12.3 y, range 8.7–19.8y), and the mean duration of follow-up was 20.8 months with a success rate of 78.2%. They concluded that compared to BMG, LMG was more feasible to harvest and had fewer complications at the donor site. Abdelhameed et al [20] studied 21 patients receiving the LMG urethroplasty who had a mean age of 36.3y and had urethral strictures after multiple operations. They achieved a success rate of 87% and confirmed the effectiveness of this method for urethral reconstruction of strictures.

We could barely find any studies about the comparison between the two grafts used in inlay urethroplasty, especially in the pre-puberty boys from a Chinese population. To evaluate the outcomes, we conducted a retrospective study of sixty-two children who received either buccal (twenty-nine) or lingual (thirty-three) mucosa graft urethroplasty in our hospital between 2012 and 2015. The ages ranged from 3.5-11y. For the outcomes of the two groups, there was no significant difference between groups in terms of age, preoperative surgery time, and the length and width of the grafts (p>0.05) (Table 2). For the lingual mucosal graft group, fistula: 2/33 (6.0%), stricture: 1/33 (3.0%), ventral curvature: 2/33(6.0%), complications: 5/33(15.0%), success rate: 28/33(84.8%), Hose score: 14.34±0.95, peak flow: 6.5 ml/s-12.0 ml/s, and mean peak flow: 9.3±0.4 ml/s. For the buccal mucosa graft group: fistula: 2/29 (6.8%), stricture: 2/29 (6.8%), ventral curvature: 1/29 (3.4%), complication rate: 5/29(17.0%), success rate: 24/29 (83.0%), Hose score: 14.28±1.03, peak flow: 6.5 ml/s-12.0 ml/s, and mean peak flow: 9.2±0.2 ml/s. There were no differences between the two groups for overall success, complication rates, the peak flow, and the Hose scores (P>0.05) (Table 3).

Our results showed that we have achieved satisfactory outcomes for the two groups. The overall successful rates of the BMG and LMG were 83.0% and 85.0%, respectively, which were similar to the 87% success rate reported by Abdelhameed et al [20] in adults in 2015. Holland [8] had reported that a total score of 14 would infer an acceptable outcome in modern hypospadias repair with the provision that the meatus is at least as long as the proximal meatus with a single urinary stream and only moderate angulation of the penile shaft. So, we achieved an acceptable or satisfactory cosmetic outcome based on the scores above. There were no significant differences between the two groups for overall successful rate and the Hose scores in our study.

We know from the literature that the lingual mucosa shares many characteristics with the urethra, such as the same embryologic origin as the urethra as well as the immunologic advantage of resistance to infection and tissue characteristics (e.g., thick epithelium, high elastic fiber content and rich vascularization), which are more obvious prior to puberty [18, 21]. These shared characteristics show that the lingual mucosa is a perfect material compared to alternative materials.

When multiple hypospadias procedures fail, 1) the failed hypospadias repairs are often associated with penile skin loss and deficient local tissues, 2) BMG are often not possible due to oral complications from a prior procedure, 3) patients undergoing failed hypospadias often have long urethral defects, and 4) the buccal mucosa grafts are not available due to frequent tissue harvesting. If buccal mucosa grafts are confronted with scarred tissues, BMG urethroplasty cannot be chosen again. So if surgeons are confronted with these problems in pre-pubertal boys, there is still a reliable graft to fix the poor repair.

It should be noted that compared to the LMG used in adults, the advantages of LMG used in children before puberty are that the tissue material is soft and thick in mucosal tissues with a better blood supply, high cell activity and survival rate. However, there are also disadvantages in that the tongues of children have a smaller volume and are harder to secure during the procedure than tongues in adults.

The length of the lingual graft in children is long enough for the defect in general, and if the urethral defect is longer, we could choose the two sides of the ventro-lateral surface of the tongue. The thickness and the articulation are related to the complications based on the outcome and the long time follow up in children. We have the relatively shorter follow up time with a good outcome, and maybe it’s a shortage of our research.

The limitations of our study include 1) a short follow-up period. A short follow-up period may have had an effect on the complication rates[22]. We had a shorter follow-up period than some reports, which would lead to a higher success rate compared to studies in the literature. We will keep on focusing on these patients to obtain long-term follow up results on the functional and cosmetic outcomes. 2) Due to the shortage of our work, it’s a pity that the post-operative complications of donor sites were not included. We will attempt to collect more details on the donor site complications in our next study. 3) We had only 62 cases in our study based on the inclusion criteria, and a larger number of pre-pubertal boys will need to be recruited to confirm the results of this study.

## Conclusion

The lingual mucosal graft and the buccal mucosa graft both achieved good outcomes, and the lingual mucosa graft made up for the shortcomings of the buccal mucosa graft and provided a reliable way to treat multiple failed hypospadias procedures in pre-pubertal boys.

## Supporting information

S1 File The conditions of the patients. (PDF)

## Abbreviations

LMG: Lingual mucosa graft urethroplasty
BMG: Buccal mucosa graft urethroplasty
