# Supplementary material for: Comparison of lingual mucosa and buccal mucosa grafts used in inlay urethroplasty in failed hypospadias of pre-pubertal boys in a Chinese group
Source: PLoS One. 2017 Aug 17;12(8):e0182803. doi: 10.1371/journal.pone.0182803 (PMC5560762; doi:10.1371/journal.pone.0182803)
Supplement: S1 File — (PDF) [file pone.0182803.s001.pdf]

Surgery	Age, y	Previous repairs(n)	Graft length (cm)	Graft width(cm)
LMG	3. 5	2	4. 5	1
LMG	3. 5	3	4	1. 3
LMG	4	3	5	1. 3
LMG	4. 5	2	5. 5	1. 1
LMG	5	3	5	1
LMG	5. 2	3	3. 5	1
LMG	5. 5	2	5. 2	1. 3
LMG	5. 5	3	4. 5	1. 4
LMG	6. 5	3	5. 5	1. 1
LMG	6. 5	3	5. 2	1
LMG	6. 5	3	5. 3	1. 2
LMG	7	3	5. 3	0. 9
LMG	7	3	5. 2	1. 2
LMG	7. 2	3	5. 3	1. 1
LMG	7. 5	3	5. 5	1. 3
LMG	7. 5	3	5. 3	1. 3
LMG	8	3	5. 4	1. 4
LMG	8	3	5	1
LMG	8. 3	3	5. 7	1. 1
LMG	8. 5	3	5. 6	0. 9
LMG	8. 5	3	5	1. 4
LMG	8. 5	3	5. 2	1
LMG	9	2	5	1. 3
LMG	9	3	5	1. 2
LMG	9. 2	2	5. 2	1. 5
LMG	9. 5	3	5	1
LMG	9. 5	3	5. 1	1. 2
LMG	10	2	5. 8	1
LMG	10	3	5	1. 2
LMG	10	3	4. 8	1
LMG	10. 5	2	5. 5	1. 3
LMG	9. 8	3	5. 5	1. 4
LMG	11	2	5	1
BMG	4	2	5. 5	1
BMG	4. 5	3	5	0. 9
BMG	5	2	4. 9	0. 9
BMG	5. 5	2	4. 5	1. 2
BMG	6. 5	3	5	1
BMG	6. 5	3	5. 1	1. 1
BMG	6. 5	2	4. 5	0. 9
BMG	6. 5	3	4. 5	1. 2
BMG	7	2	5	1. 1
BMG	7	3	5. 5	0. 9
BMG	7. 5	2	5	0. 8
BMG	7. 5	3	5	1. 3
BMG	7. 5	3	5	1. 2
BMG	8	3	4. 5	1. 1
BMG	8. 2	2	5	0. 9
BMG	8. 5	2	4. 5	1. 3
BMG	9	3	4. 9	1. 1
BMG	9	3	5. 2	0. 8
BMG	9. 2	2	5	1
BMG	5	3	4. 9	0. 9

BMG	7.5	3	5	1.3
BMG	8	2	5	1.5
BMG	7.5	2	5.2	1.1
BMG	6	2	5.5	1
BMG	6.5	3	5	1.2
BMG	8.2	2	5.2	1.5
BMG	7	3	5.1	1
BMG	8.2	2	4.6	0.9
BMG	7	3	5.2	1.2

Follow-up time（m）	The peak flow(ml/s)	hose score	Fistula	Stricture
6	9	14	NO	NO
10	10	15	NO	NO
6	9.2	15	NO	NO
7.5	9.5	16	NO	NO
7	8.9	16	NO	NO
9.2	9	13	NO	NO
7.5	10	14	NO	NO
8.5	9.5	14	NO	NO
9	9.7	13	YES	NO
11	9	16	NO	NO
8	9.8	14	NO	NO
8.5	9	16	NO	NO
9	9.7	15	NO	NO
10	9.5	15	NO	NO
7.5	8.5	14	NO	YES
9.5	9.5	15	NO	NO
8	9.1	13	NO	NO
8.5	8.9	15	NO	NO
7.5	8.4	14	YES	NO
9	9.5	15	NO	NO
10	9	14	NO	NO
7	8.8	15	NO	NO
8	9.5	13	NO	NO
8.5	9.3	14	NO	NO
6.5	10.1	14	NO	NO
8.3	9.3	15	NO	NO
9	9.3	13	NO	NO
7	9.5	13	NO	NO
8.2	9	15	NO	NO
9.5	9	14	NO	NO
8	9.6	14	NO	NO
8.3	9.5	14	NO	NO
8.5	9.1	13	NO	NO
6.8	8.5	14	YES	NO
7	9.3	15	NO	NO
7.8	9	13	NO	NO
8	9.4	14	NO	NO
7.5	8.9	13	NO	YES
8	9	14	NO	NO
7.8	9.5	16	NO	NO
8	9.2	14	NO	NO
8.5	9	15	NO	NO
7.8	9.6	16	NO	NO
7.9	9.1	13	NO	NO
8.1	9	13	NO	NO
8.5	9.4	14	NO	NO
8	9.4	14	NO	NO
7.9	9.3	15	NO	NO
7.5	9.5	13	NO	NO
7.8	9	16	NO	NO
7	9.5	15	NO	NO
8.8	9	14	NO	YES
7	9.3	15	NO	NO

8.5	10	13	YES	NO
8.8	9.2	14	NO	NO
7.5	9	14	NO	NO
8	9.5	13	NO	NO
8.1	9.2	15	NO	NO
8	9	13	NO	NO
8.5	9.1	15	NO	NO
8.5	9.4	16	NO	NO
9.2	9.6	15	NO	NO

Ventral curvature	diverticula	glans dehiscence	skin reoperation with some bleeding
NO	NO	NO	NO
NO	NO	NO	NO
NO	NO	NO	NO
NO	NO	NO	NO
NO	NO	NO	NO
NO	NO	NO	NO
NO	NO	NO	NO
NO	NO	NO	NO
NO	NO	NO	NO
YES	NO	NO	NO
NO	NO	NO	NO
NO	NO	NO	NO
NO	NO	NO	NO
NO	NO	NO	NO
NO	NO	NO	NO
NO	NO	NO	NO
YES	NO	NO	NO
NO	NO	NO	NO
NO	NO	NO	NO
NO	NO	NO	NO
NO	NO	NO	NO
NO	NO	NO	NO
NO	NO	NO	NO
NO	NO	NO	NO
NO	NO	NO	NO
NO	NO	NO	NO
NO	NO	NO	NO
NO	NO	NO	NO
NO	NO	NO	NO
NO	NO	NO	NO
NO	NO	NO	NO
NO	NO	NO	NO
NO	NO	NO	NO
NO	NO	NO	NO
NO	NO	NO	NO
NO	NO	NO	NO
NO	NO	NO	NO
NO	NO	NO	NO
NO	NO	NO	NO
NO	NO	NO	NO
NO	NO	NO	NO
NO	NO	NO	NO
NO	NO	NO	NO
NO	NO	NO	NO
YES	NO	NO	NO
NO	NO	NO	NO
NO	NO	NO	NO
NO	NO	NO	NO
NO	NO	NO	NO
NO	NO	NO	NO
NO	NO	NO	NO
NO	NO	NO	NO
NO	NO	NO	NO

NO	NO	NO	NO
NO	NO	NO	NO
NO	NO	NO	NO
NO	NO	NO	NO
NO	NO	NO	NO
NO	NO	NO	NO
NO	NO	NO	NO
NO	NO	NO	NO
NO	NO	NO	NO

a loss of stent
NO
NO
NO
NO
NO
NO
NO
NO
NO
NO
NO
NO
NO
NO
NO
NO
NO
NO
NO
NO
NO
NO
NO
NO
NO
NO
NO
NO
NO
NO
NO
NO
NO
NO
NO
NO
NO
NO
NO
NO
NO
NO
NO
NO
NO
NO
NO
NO
NO
NO
NO
NO
NO

NO
NO
NO
NO
NO
NO
NO
NO
NO
